# A Community-Based Workshop on Addressing Dementia-Related Stigma: First Insights From a Rural Community

**DOI:** 10.1093/geroni/igaa057.3236

**Published:** 2020-12-16

**Authors:** Juanita Bacsu, Marc Viger, Shanthi Johnson, Megan O’Connell

**Affiliations:** 1 University of Regina, Regina, Saskatchewan, Canada; 2 University of Saskatchewan, Saskatoon, Saskatchewan, Canada; 3 University of Alberta, Edmonton, Alberta, Canada

## Abstract

Dementia-related stigma can delay early dementia diagnosis and lead to social isolation, depression, and suicide. Despite this knowledge, few studies identify strategies to reduce dementia-related stigma. This late-breaker poster begins to address this gap by showcasing the educational components of a community-based workshop to share study findings on reducing dementia-related stigma in rural communities. Guided by solutions-focused theory, semi-structured interviews were conducted with 18 seniors including family members, friends, caregivers and people affected by dementia and other forms of cognitive impairment in rural Saskatchewan, Canada. A focus group was conducted with 7 rural community leaders. The interview and focus group transcripts were analyzed using thematic analysis. Based on the interview and focus group findings, educational components of the workshop included: a dementia definition, different dementia types, warning signs/symptoms, risk reduction strategies, and information on dementia-related stigma and myths. Several strategies to reduce stigma were identified ranging from hosting inter-generational programs to inviting guest speakers with dementia. This study was found to be beneficial for improving knowledge, attitudes, comfort levels, and awareness of dementia. Additional research is needed to develop, implement, and evaluate interventions to reduce dementia-related stigma in different cultures and contexts.

